# Prescribing Under Uncertainty: The Hidden Psychology of Psychiatric Decision-Making

**DOI:** 10.62641/aep.v54i3.2271

**Published:** 2026-06-15

**Authors:** Carlos De las Cuevas

**Affiliations:** ^1^Department of Internal Medicine, Dermatology and Psychiatry, School of Medicine, University of La Laguna, 38071 San Cristóbal de La Laguna, Canary Islands, Spain; ^2^Instituto Universitario de Neurociencia (IUNE), University of La Laguna, 38071 San Cristóbal de La Laguna, Canary Islands, Spain

## Introduction

Psychiatric practice is inherently shaped by uncertainty. Unlike many areas of medicine, it often operates without definitive biomarkers, relying instead on probabilistic diagnoses and trajectories that remain difficult to predict. As a result, clinical decisions are frequently made under conditions of ambiguity, where the boundaries between disorder and distress, treatment and adaptation, or benefit and harm are not always clearly defined [1, 2].

In this context, psychopharmacological prescribing is typically framed as a rational, evidence-based response to patient need. However, this perspective may overlook a critical and underexamined dimension of clinical decision-making: the psychological experience of the clinician. Evidence from cognitive psychology and medical decision-making suggests that judgments under uncertainty are not guided solely by evidence, but are also shaped by cognitive biases, affective responses, and individual tolerance for ambiguity [3, 4]. In particular, intolerance of uncertainty has been identified as a key factor influencing how individuals perceive and respond to ambiguous situations [1].

Clinical decision-making in psychiatry is often more complex than a purely evidence-based model suggests, and studies indicate that shared decision-making remains inconsistently implemented in routine care [5]. In addition, psychiatric patients frequently report variable levels of involvement in treatment decisions, underscoring the ambiguity that often surrounds decision-making in this field [6].

This raises a critical question: to what extent might prescribing function not only as an intervention directed at the patient, but also as a response to the clinician’s own uncertainty? When diagnostic clarity is limited and outcomes remain unpredictable, prescribing may provide a sense of direction and provisional resolution, whereas deferring treatment requires the capacity to tolerate ambiguity over time.

Importantly, this phenomenon should not be reduced to individual shortcomings. Rather, it emerges from the interaction between cognitive tendencies, emotional responses, and structural pressures inherent to contemporary healthcare systems, including time constraints, patient expectations, and medico-legal considerations [7]. Psychiatry, given its intrinsic epistemic uncertainty, may represent a particularly fertile context for these dynamics.

In this article, we argue that the medicalisation of uncertainty constitutes an underrecognised driver of psychopharmacological prescribing. By shifting the analytical lens from patient pathology to clinician experience, we examine the psychological and structural factors that shape decision-making under uncertainty, explore their clinical consequences, and propose a reframing of uncertainty tolerance as a core clinical competence in psychiatric practice.

## Shifting the Lens: From Patient Need to Clinician Experience

Clinical decision-making in psychiatry is traditionally conceptualised as a patient-centred process: symptoms are assessed, diagnoses are formulated, and treatments are selected according to the best available evidence. Within this framework, prescribing is understood as a response to clinical need. However, this perspective may be incomplete, as it tends to overlook the extent to which the clinician’s own cognitive and emotional processes shape therapeutic decisions.

Research in cognitive psychology and medical reasoning has consistently shown that clinical judgments are influenced not only by analytical reasoning but also by heuristics, biases, and affective states [4, 8]. These influences become particularly salient under conditions of uncertainty, where information is incomplete and outcomes remain unpredictable—circumstances that are common in psychiatric practice.

In such situations, the clinician is confronted not only with a clinical problem but also with an internal state of doubt. This state is both cognitively demanding and emotionally aversive, creating a natural tendency toward resolution. Prescribing may therefore serve a dual function: as an intervention directed at the patient and as a means of reducing the clinician’s own uncertainty. By introducing a course of action, it provides a sense of direction, agency, and provisional closure.

This perspective does not imply that prescribing under uncertainty is inappropriate. Rather, it suggests that the motivations underlying clinical decisions are more complex than is often acknowledged. Affective influences on decision-making have been shown to shape risk perception and action selection, often outside of conscious awareness [9], while the need for cognitive closure drives the preference for definitive answers over sustained ambiguity [10]. Within this framework, deferring action—despite being clinically reasonable—may be experienced as psychologically uncomfortable, whereas prescribing offers a form of resolution even in the absence of clear evidence.

Recognising the role of clinician experience does not undermine clinical judgment, but rather enriches it. By making these processes explicit, it becomes possible to better understand how uncertainty is managed in practice—and how, at times, it may be transformed into action.

## The Psychology of Prescribing Under Uncertainty

Decision-making under uncertainty is not a neutral process. It is shaped by interacting cognitive and affective mechanisms that influence how ambiguity is perceived, tolerated, and resolved. In clinical practice, these processes often operate implicitly, yet they play a central role in determining whether uncertainty is sustained or converted into action.

A key construct in this context is intolerance of uncertainty (IU), defined as the tendency to perceive uncertain situations as stressful or unacceptable [1]. Higher levels of IU are associated with a preference for rapid resolution, favouring actions that reduce ambiguity in the short term, even when their long-term benefit is uncertain. For clinicians, uncertainty is therefore not only informational but also experiential—something to be managed rather than merely understood.

This tendency is reinforced by action bias, the inclination to favour intervention over inaction in situations of uncertainty or perceived risk [11]. In psychiatry, where clinical evolution is often slow and diagnostic boundaries remain fluid, action bias may lower the threshold for initiating or escalating treatment. Prescribing, in this context, offers a behavioural pathway through which uncertainty can be reduced.

At a cognitive level, the need for closure further contributes to this dynamic. The desire for definitive answers promotes early diagnostic and therapeutic decisions, which can prematurely stabilise interpretations and limit reconsideration of alternatives [10]. Prescribing may thus function as a form of cognitive closure, transforming an open-ended situation into a structured and manageable one.

These processes are closely intertwined with affective factors. Uncertainty is inherently aversive, generating anxiety and a heightened sense of responsibility. The anticipation of potential negative outcomes—particularly in vulnerable patients—may lead clinicians to act in order to avoid future regret [12]. This anticipated regret amplifies the perceived cost of inaction, further shifting decision-making toward intervention.

**Fig. 1.**
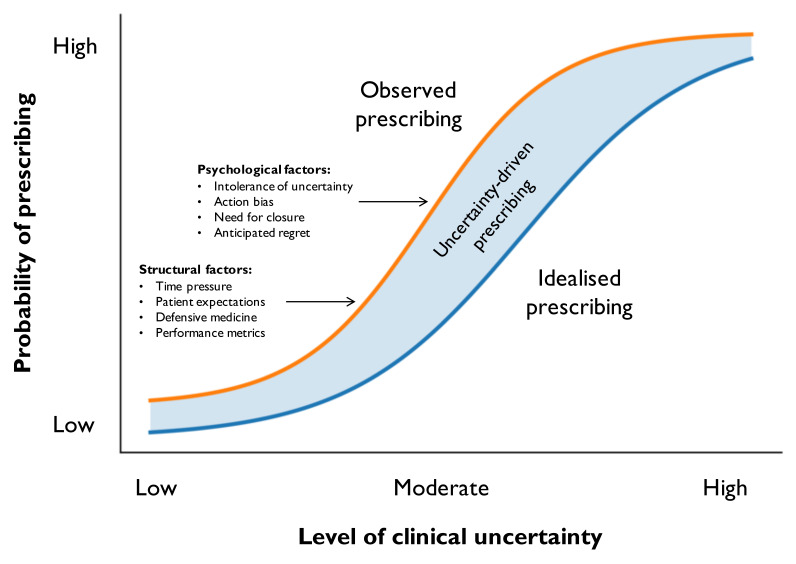
**Conceptual model of the shift from uncertainty to action in psychiatric prescribing**.

Importantly, these mechanisms do not operate in isolation but reinforce one another. Intolerance of uncertainty increases the discomfort associated with ambiguity; action bias provides a means of alleviating it; need for closure favours rapid resolution; and anticipated regret heightens the perceived risks of inaction. Together, they create a cognitive-affective environment in which prescribing becomes not only a clinical option but a psychologically compelling response (Fig. 1).

## Legend

This conceptual model illustrates the association between clinical uncertainty and the probability of prescribing. Both curves follow a sigmoid (S-shaped) trajectory, capturing the non-linear nature of this association. The lower curve represents an idealised scenario in which prescribing is determined primarily by patient need, with intervention increasing gradually as clinical clarity emerges. The upper curve reflects real-world practice, where psychological and structural factors shift the threshold toward earlier and more frequent intervention.

The area between the curves represents prescribing driven predominantly by the need to reduce uncertainty rather than by clear clinical indication. This shift is influenced by interacting cognitive and affective mechanisms—such as intolerance of uncertainty, action bias, need for closure, and anticipated regret—as well as by systemic factors including time pressure, patient expectations, and medico-legal concerns.

Recognising these processes does not imply that prescribing under uncertainty is inappropriate. Rather, it highlights that such decisions are embedded in a broader psychological context that deserves explicit consideration. Making these mechanisms visible may help clinicians to distinguish more clearly between actions driven primarily by patient need and those influenced, at least in part, by the desire to resolve uncertainty.

## Structural Drivers: Why the System Favors Action

While cognitive and affective mechanisms shape how clinicians respond to uncertainty, these processes are embedded within healthcare systems that consistently favour action over inaction. The tendency to prescribe under uncertainty is therefore not only psychologically driven but structurally reinforced.

One of the most immediate factors is time pressure. High patient volumes and brief consultations limit opportunities for longitudinal assessment or sustained exploration of ambiguity. Under such conditions, prescribing provides a practical means of advancing clinical encounters, whereas deferring intervention requires time that is often unavailable. Structural constraints thus compress clinical reasoning, favouring decisions that offer immediate direction over those that preserve uncertainty.

Patient expectations further contribute to this dynamic. Many individuals seeking psychiatric care anticipate a clear diagnosis and an active intervention, frequently in the form of medication. Within this context, a watchful waiting approach may be perceived as insufficient, even when clinically appropriate. Prescribing can therefore function not only as a therapeutic decision but also as a communicative act that aligns with expectations and reinforces the therapeutic alliance.

A broader biomedical culture of intervention also shapes clinical behaviour. Contemporary medicine is largely oriented toward active treatment, with inaction often implicitly associated with passivity or neglect. This orientation can lower the threshold for intervention, particularly in psychiatry, where distinctions between pathology and normal variation are often uncertain. As a result, ambiguity is more readily translated into treatment than into observation.

Medico-legal considerations introduce an additional layer. In environments where clinical decisions may be retrospectively scrutinised, the perceived risks of under-treatment may outweigh those of over-treatment. Prescribing can thus be understood as a form of professional self-protection, consistent with patterns of defensive medicine [13].

Finally, healthcare systems increasingly rely on metrics and performance indicators that privilege measurable actions. Treatment initiation, symptom reduction, and service throughput are more easily quantified than the appropriateness of restraint or the quality of uncertainty management. Consequently, prescribing becomes visible and valued, whereas non-intervention—however appropriate—remains largely unrecognised.

Taken together, these structural factors create an environment in which action is not only psychologically appealing but institutionally supported. The medicalisation of uncertainty should therefore be understood not simply as an individual tendency, but as an emergent property of systems that systematically lower the threshold for intervention.

## Clinical Consequences: When Action Becomes Overaction

The convergence of psychological tendencies and structural pressures creates a clinical environment in which action is not only favoured but may become excessive. While prescribing under uncertainty is often well-intentioned and sometimes appropriate, its cumulative effects can extend beyond what is supported by evidence or required by patient need.

One immediate consequence is **overprescription**, particularly in cases of mild, transient, or diagnostically ambiguous presentations. In such situations, pharmacological treatment may be initiated as a precautionary measure or as a way of responding to clinical uncertainty, even when non-pharmacological approaches or watchful waiting would be reasonable alternatives. This pattern reflects broader concerns about overdiagnosis and overtreatment, where expanding diagnostic boundaries and lowered thresholds for intervention increase the likelihood of limited-benefit care [14, 15].

A related phenomenon is the development of polypharmacy. When uncertainty persists despite initial treatment—or when new ambiguities emerge—there may be a tendency to add or modify medications rather than reconsider the underlying clinical formulation. Each additional prescription can be understood as an attempt to address residual uncertainty, leading to progressively more complex treatment regimens. In turn, polypharmacy introduces risks of drug interactions, adverse effects, and increasing difficulty in attributing clinical outcomes to specific interventions.

The difficulty of treatment discontinuation represents a further consequence. While initiating medication may reduce immediate uncertainty, discontinuing it often reintroduces it: questions about relapse, prior effectiveness, or ongoing necessity may discourage withdrawal. As a result, treatments are frequently maintained by default, contributing to prolonged exposure without systematic reassessment of benefit.

More broadly, these dynamics contribute to a pharmacological framing of distress, in which uncertainty is preferentially managed through medication. Situations that might otherwise be understood in psychosocial or developmental terms may instead be interpreted within a biomedical framework, narrowing the range of therapeutic responses.

These patterns do not reflect inappropriate practice in a simplistic sense, but rather the accumulation of individually reasonable decisions made under uncertainty. However, taken together, they point toward a state of overaction, in which the overall intensity of intervention exceeds what is proportionate to the clinical context. Recognising this shift invites reflection not only on what is prescribed, but on why and under which conditions prescribing occurs.

## Psychiatry as a Special Case of Uncertainty

Uncertainty is a fundamental feature of all medical practice, but its nature and extent vary across disciplines. Psychiatry occupies a distinctive position, characterised by a form of epistemic uncertainty that is both pervasive and structurally embedded. This reflects not a lack of scientific rigour, but the complexity of its subject matter and the current limits of available knowledge.

A central feature is the absence of definitive biological markers for most psychiatric conditions. Diagnosis relies primarily on clinical observation, patient-reported experience, and evolving symptom patterns, resulting in categories that are inherently probabilistic and whose boundaries remain fluid [16]. This diagnostic indeterminacy is further compounded by heterogeneous trajectories and variable treatment responses, where similar presentations may follow markedly different courses over time.

In addition, psychiatric assessment is fundamentally interpretative, requiring the integration of narrative, context, and meaning rather than reliance on stable external measures. While this enables a nuanced understanding of individual experience, it also amplifies variability and limits the possibility of definitive conclusions at any given time.

Taken together, these features situate psychiatry at the intersection of scientific knowledge and interpretative practice, where uncertainty is not an exception but a condition of work. In this context, the tendency to translate uncertainty into action—particularly through prescribing—may become especially pronounced.

Recognising this does not diminish the discipline, but rather reframes its demands. If uncertainty is intrinsic to psychiatric practice, then clinical expertise must include not only the capacity to act, but also the capacity to tolerate and manage ambiguity without prematurely resolving it.

## Toward a Different Model: Tolerating Uncertainty as Clinical Skill

If uncertainty is an inherent and irreducible component of psychiatric practice, the challenge is not to eliminate it, but to engage with it more effectively. The preceding analysis suggests that uncertainty is often transformed into action—particularly through prescribing—in ways that reflect not only patient need, but also clinician experience and systemic pressures. Addressing this requires a shift in how uncertainty is understood within clinical work.

A first step is to recognise tolerance of uncertainty as a core clinical competence. While medical training emphasises diagnostic accuracy and treatment selection, less attention is given to the capacity to sustain ambiguity, resist premature closure, and make decisions that do not immediately resolve uncertainty. Yet these capacities are central to sound clinical judgment, particularly in psychiatry.

This has implications for everyday practice. Watchful waiting and temporal observation should not be seen as passive alternatives, but as active strategies that allow clinical meaning to emerge over time. Similarly, uncertainty can be more explicitly shared with patients, not as a limitation of care, but as a reflection of the current state of knowledge. In this context, shared decision-making becomes not only a matter of choice, but of jointly engaging with what cannot yet be fully determined [17]. However, evidence suggests that shared decision-making remains inconsistently implemented in psychiatric practice, and that patients frequently experience significant decisional conflict when confronted with treatment options, reflecting the inherent ambiguity of clinical decisions in this field [5, 6].

At a broader level, there is a need to better align healthcare systems with the realities of uncertainty. Structures that prioritise immediacy, action, and measurable outputs may inadvertently discourage clinical restraint. Recognising the value of time, reflection, and non-intervention is therefore essential if uncertainty is to be managed rather than prematurely resolved.

The aim is not to reduce prescribing, but to recalibrate the threshold at which it occurs, ensuring that intervention reflects patient need more than the pressures surrounding clinical decision-making. In this sense, tolerating uncertainty is not a passive stance, but a form of clinical discipline requiring judgment, restraint, and reflexivity.

Psychiatry operates at the limits of what can be known with certainty. Within this landscape, the capacity to act is indispensable—but so too is the capacity to refrain. The question, therefore, may not only be what to prescribe, but when uncertainty should be sustained rather than resolved through action.

## Availability of Data and Materials

Not applicable.
